# Bovine embryo production *in vitro*: evolution of culture media and commercial perspectives

**DOI:** 10.1590/1984-3143-AR2024-0051

**Published:** 2024-09-23

**Authors:** Rebecca L. Krisher, Jason R. Herrick

**Affiliations:** 1 Genus plc, Genus Research and Development, DeForest, WI, USA; 2 Omaha’s Henry Doorly Zoo and Aquarium, Omaha, NE, USA

**Keywords:** embryo culture, *in vitro* embryo production, culture media, embryo metabolism

## Abstract

*In vitro* produced embryos exhibit lower viability compared to their *in vivo* counterparts. Mammalian preimplantation embryos have the ability to reach the blastocyst stage in diverse culture media, showcasing considerable metabolic adaptability, which complicates the identification of optimal developmental conditions. Despite embryos successfully progressing to the blastocyst stage, adaptation to suboptimal culture environments may jeopardize blastocyst viability, cryotolerance, and implantation potential. Enhancing our capacity to support preimplantation embryonic development *in vitro* requires a deeper understanding of fundamental embryo physiology, including preferred metabolic substrates and pathways utilized by high-quality embryos. Armed with this knowledge, it becomes achievable to optimize culture conditions to support normal, *in vivo*-like embryo physiology, mitigate adaptive stress, and enhance viability. The objective of this review is to summarize the evolution of culture media for bovine embryos, highlighting significant milestones and remaining challenges.

## Introduction

While advancements have been made in bovine oocyte maturation and embryo culture over the past decades, *in vitro* embryo technologies capable of producing embryos with similar viability as those developing *in vivo* have remained elusive. There is still much to unravel regarding the *in vitro* requirements of oocytes and embryos to facilitate successful development and the production of healthy offspring. Exposure of embryos to suboptimal culture conditions, resulting in altered embryo metabolism, not only leads to decreased blastocyst formation and reduced embryo viability, but also negatively impacts the maintenance of pregnancy, fetal growth, and health of offspring (Farin et al., 2001). The metabolic adaptability of embryos is remarkable, but it comes at considerable cost. Therefore, to mitigate the adaptive stress leading to poor embryo quality, diminished pregnancy potential, and adverse health outcomes in future offspring, it is imperative that embryo culture conditions support normal embryo physiology.

Oocyte maturation *in vitro* is a critical component of *in vitro* embryo production, but it will not be covered here. Development of maturation medium that better supports oocyte developmental competence is worthy of an independent review. The reader is directed to other manuscripts for treatment of this topic (Krisher, 2004, 2013; Labrecque and Sirard, 2014; Dumesic et al., 2015; Lonergan and Fair, 2016; Aguila et al., 2020; Marei and Leroy, 2022; Fair and Lonergan, 2023).

## Commercial application of bovine *in vitro* embryo production

Each year, the Data Retrieval Committee of the International Embryo Technology Society (IETS) gathers, organizes, and publishes statistics describing the embryo industry in farm animal species. The most recent data available for the calendar year 2022 show that the global bovine embryo industry continues to grow (Viana, 2023). However, the actual growth rate between 2021 and 2022 (5.5%) was decreased compared to that observed between 2020 and 2021 (25.6%). For *in vitro* produced (IVP) embryos specifically, from 2020 to 2021 the number of embryos produced grew by 31.5%, whereas between 2021 and 2022, this growth rate dropped to 6.3%. Nonetheless, almost 1.5 million bovine embryos were transferred globally in 2022. Cryopreservation is also a growing trend in cattle embryo technologies, with almost half of all cattle embryo transfers utilizing frozen embryos. In vivo derived embryos are more commonly cryopreserved (65%) than IVP embryos (44%), due in part to reduced cryotolerance of IVP embryos particularly when slow freezing/direct transfer is used versus vitrification. This difference in cryotolerance has implications for how or even if IVP embryos are used for export. In general, embryo technologies are utilized in 28% of countries worldwide, with 95% of the reported embryos being specifically cattle embryos. Notably, more than 80% of all bovine embryos produced globally are *in vitro* derived, and *in vitro* derived embryos continue to drive growth in commercial embryo technologies. Based on the sales of embryo transfer materials, the data retrieval committee suggests that these numbers are in fact an underestimate and that the actual number of embryos produced *in vitro* in 2022 was likely more than 2 million globally. Indeed, for the last decade *in vitro* technologies have been supplanting superovulation and collection of *in vivo* embryos as the preferred method for cattle embryo production. In 2022, seven out of the top 10 embryo producing countries reported a higher number of IVP embryos compared to *in vivo* derived (IVD) embryos.

What accounts for the rapid increase in the adoption of *in vitro* embryo technologies? There is mounting pressure on animal agriculture to produce more protein, both milk and meat, for a growing world population. At the same time, producers must be mindful of sustainability goals and expectations from consumers. Embryo technologies can help to achieve both objectives. Genetically superior animals, identified by genotyping, are more efficient in their production of protein. The use of semen in conventional AI allows bulls of high genetic merit to contribute more to the next generation of production animals, but male genetics are only half the equation. Using embryos accounts for both male and female genetics, making genetic progress much faster (Sirard, 2018). A valuable female can produce significantly more embryos over her lifetime when ovum pick up and *in vitro* fertilization are used to produce embryos, versus superovulation and embryo flushing. Oocytes can even be collected from prepubertal and pregnant females. Importantly, using ovum pick up in calves via laparoscopy and in pre-pubertal heifers can significantly shorten generation interval, resulting in even faster genetic gain. This approach enables the most genetically superior animals to make the greatest contribution to the succeeding generation, leading to swift improvements in production efficiency and ultimately enhancing the sustainability of animal agriculture overall. Currently, the higher cost of embryos relative to semen is the main roadblock to even greater implementation of embryo technologies, given that pregnancy rates are roughly equal between the two technologies. Efforts to automate embryo production to drive down costs while increasing efficiency, quality, and scale are currently being investigated. Relevant to both human and bovine embryo technology, semi-automated vitrification systems have been reported and used in clinical trials (Wang et al., 2024; Roy et al., 2014; Hajek et al., 2021; Barberet et al., 2022).

## Challenges of *in vitro* fertilization technology

**Figure 1:**
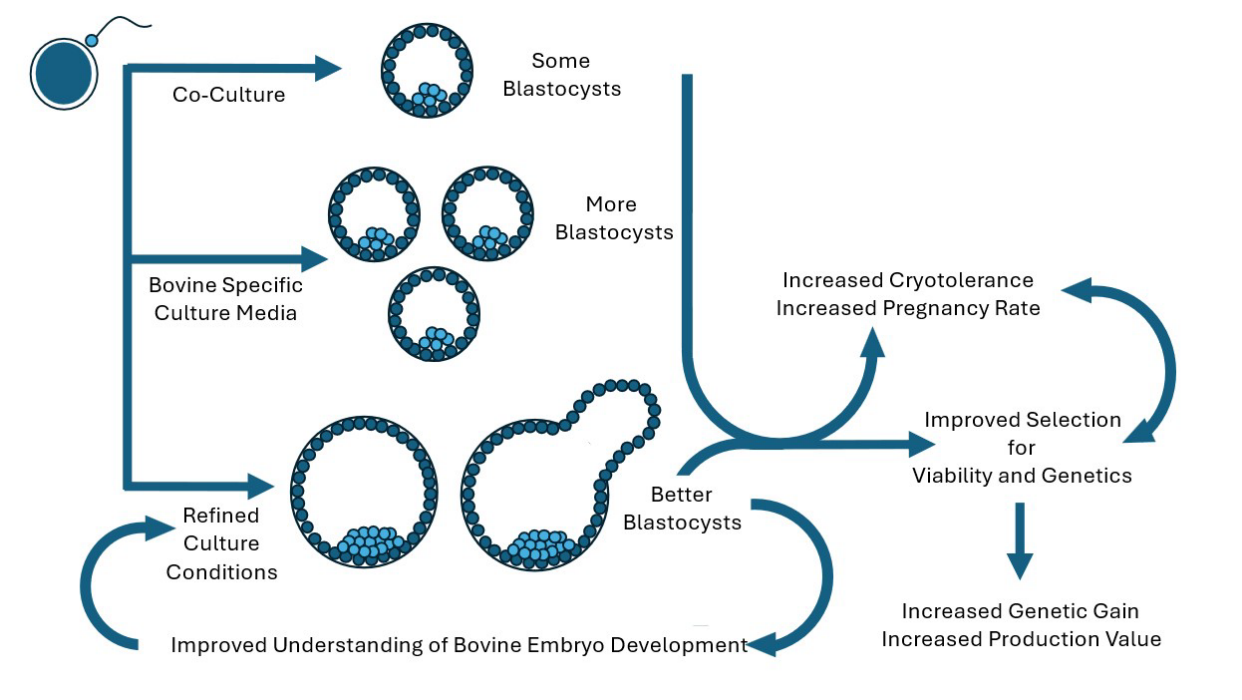
A better understanding of embryo biology informs the development of more effective culture media. Improved media produce embryos that are more *in vivo-*like, which further advance our understanding of embryo biology, improve cryotolerance and the outcome of embryo transfer, and ultimately result in increased genetic gain, increased production value and producer profit, and a more sustainable industry.

*In vitro* embryo production, despite its widespread commercial application, remains an inefficient process. This inefficiency significantly contributes to the high cost per embryo, limiting adoption for many producers. The largest inefficiency in the system is the loss between oocytes recovered at ovum pick up and transferrable quality embryos produced following in vitro fertilization (IVF). Only about 25% of all oocytes collected become good quality embryos, and this number is even lower if sexed semen is used for fertilization. Another large loss occurs following embryo transfer, as less than 50% of good quality embryos result in pregnancy. Early embryonic losses before day 60, as well as additional losses to abortion or stillborn calves, contribute as well. Overall, fewer than 10 calves are born for every 100 viable oocytes collected. Improvement in the system would mean that oocytes could be collected from fewer, more genetically valuable donors, requiring less farm and laboratory labor to reduce cost and advance genetic gain (Figure 1). However, despite concentrated efforts to improve embryo culture media over the last 20 years, we have seen only minimal improvement in embryo production efficiencies and embryo quality.

## Embryo metabolism and the evolution of culture media

The identification of a suitable combination of nutrients, including appropriate concentrations and timing of provision, was pivotal to the formulation of successful embryo culture media. The pioneering investigations into embryo culture utilized murine embryos, which largely rely on carbohydrate metabolism to support development (Brinster, 1965; Biggers et al., 1967). Embryos at the early cleavage stages primarily utilize pyruvate and lactate, whereas in later stages, there is a transition to reliance on glucose metabolism through glycolysis to support formation and expansion of the blastocyst (Gardner and Leese, 1987; Gardner et al., 2001). Murine embryos could develop to the blastocyst stage in simple salt solutions with glucose, lactate, and pyruvate as the only energy sources, usually in the presence of albumin (Whitten and Biggers, 1968). Although the general pattern of carbohydrate metabolism is similar for bovine embryos, with the early cleavage stages primarily reliant on pyruvate oxidation and glycolysis becoming the dominant pathway after the morula stage (Rieger et al., 1992a, 1992b; Kim et al., 1993; Thompson et al., 1996; Krisher et al., 1999), early culture media developed for the mouse were not effective for culturing bovine embryos. Therefore, the first embryo culture systems for bovine embryos utilized media designed for somatic cell culture and often relied heavily on support cells in a co-culture environment. Co-culture was able to overcome the 8-16 cell block in bovine embryos, as well as improve the quantity and quality of blastocysts produced (Ellington et al., 1990; Goto et al., 1992; Voelkel and Hu, 1992; Thomas and Seidel, 1993; Abe and Hoshi, 1997; Krisher et al., 1998). Similar positive effects were observed using cell culture conditioned medium (Eyestone et al., 1991; Hernandez-Ledezma et al., 1993; Vansteenbrugge et al., 1994). It was hypothesized that somatic cells removed inhibitory substances from the culture media, which at that time was sub-optimal. However, the complexity and undefined nature of co-culture and conditioned media systems made it virtually impossible to study the metabolic requirements of preimplantation bovine embryos *in vitro*.

Although many media have been independently developed, three media are the backbone formulations of most bovine culture media used today: synthetic oviductal fluid (SOF), Charles Rosenkrans medium with amino acids (CR1AA), and potassium simplex optimized medium (KSOM), each developed using slightly different approaches. Synthetic oviductal fluid has been a frequently utilized medium for culturing bovine embryos *in vitro* for more than 20 years. Initially, SOF was developed through the biochemical analysis of ovine oviductal fluid (Tervit et al., 1972). The strategy of mimicking the *in vivo* environment has been described as the ‘back to nature’ approach (Leese, 1998). Another extension of mimicking the in vivo environment was the concept of sequential media specifically devised to cater to the evolving needs of the embryo during its developmental journey, with one medium for early cleavage stage embryos and another for compaction and blastocyst formation (Gardner and Lane, 1997). A variety of single-step and sequential versions of SOF have been developed, which include the supplementation of amino acids (Gardner et al., 1994; Steeves and Gardner, 1999), incorporation of citrate (Keskintepe et al., 1995), the exclusion of glucose (Takahashi and First, 1992), and the inclusion of EDTA for the first 72 hours of the culture period (Gardner et al., 2000). One of the other primary strategies for culture medium design is to provide the embryo with all the nutrients it may need in a single medium, that may or may not be refreshed during the culture period. The rationale behind this strategy is to let the embryo select which nutrients it utilizes for development from those provided and is often described as ‘let the embryo choose’ (Biggers, 1998; Biggers and Racowsky, 2002). Empirical evaluations of the effects of specific carbohydrates, amino acids, and vitamins led to the formulation of CR1AA (Rosenkrans et al., 1993; Rosenkrans and First, 1994). A systematic approach known as simplex optimization was used to develop KSOM (potassium simplex optimized medium), originally designed to overcome the 2-cell block in mouse embryos and allow development to the blastocyst stage (Lawitts and Biggers, 1991, 1992; Erbach et al., 1994). This medium was quickly adapted for bovine embryo culture (Liu and Foote, 1995, 1996, 1997; Moreira et al., 2002). There are advantages and disadvantages to each type of system, and one system has not proven to be superior to the other (Macklon et al., 2002; Biggers and Summers, 2008; Machtinger and Racowsky, 2012; Quinn, 2012; Swain et al., 2016; Swain, 2019). Typically, SOF is used as a sequential medium (Matsuyama et al., 1993; Gardner et al., 2000; Gandhi et al., 2000), whereas CR1AA and KSOM are often used as a single step medium. Use of these two different systems has been debated for more than ten years, and choice is often dictated by what is currently in fashion, or what is most convenient for laboratory workflow.

Once media were available that would effectively support the development of bovine blastocysts *in vitro*, an increased emphasis was placed on not just producing blastocysts, but on the quality/viability of those blastocysts and their potential to produce healthy offspring. The conditions under which embryos are cultured impacts their ability to metabolize glucose (Du and Wales, 1993a, b; Rieger et al., 1995). Because the uptake and utilization of glucose are intricately linked to the developmental competency of embryos and likely mirror the energy needs of the embryo (Renard et al., 1980; Gardner and Leese, 1987), culture media choice and optimal laboratory practices are critical to produce high quality, viable blastocysts. Embryos cultured in oviductal cell-conditioned medium exhibited heightened glucose metabolism rates, but lower cell counts and delayed development, indicating that elevated glucose metabolism rates might correlate with diminished embryo viability (Rieger et al., 1995). An increased rate of glucose metabolism at the blastocyst stage has also been associated with reduced embryo viability in mice (Lane and Gardner, 1996).

Once metabolic changes were linked to viability the next step in the evolution of culture media was to provide a specific set of substrates to modulate the metabolism of *in vitro*-produced embryos to closely emulate their more viable counterparts produced *in vivo*. One such strategy is based on the “Quiet Embryo Hypothesis”, which states that viable embryos, exhibit reduced oxidative phosphorylation activity and consequently consume less oxygen because they require less energy to repair stress-induced damage (Leese, 2002; Baumann et al., 2007; Leese et al., 2007, 2008). Another hypothesis suggests that embryo metabolism might resemble that observed in rapidly proliferating cancer cells, a metabolic phenomenon referred to as the Warburg effect (Krisher and Prather, 2012; Smith and Sturmey, 2013). In this model, the most viable embryo is not necessarily the one exhibiting the lowest activity within a particular pathway, but rather the embryo metabolizing specific substrates through the most suitable pathway(s). As the focus has shifted to an evaluation of overall embryo metabolic activity thru multiple pathways, the importance of fatty acids and amino acids as substrates has become apparent.

Somewhat unexpectedly, sheep, pig, and cow embryos can thrive successfully with or without glucose in the culture medium (Tervit et al., 1972; Petters et al., 1990; Takahashi and First, 1992; Thompson et al., 1992; Rosenkrans and First, 1994; Steeves and Gardner, 1999; Gandhi et al., 2000). It is evident that embryos in these species are capable of utilizing endogenous fatty acids and/or amino acids for energy production even in the absence of carbohydrates. Mammalian embryos utilize fatty acids as an energy source (Ferguson and Leese, 2006; Sturmey et al., 2006, 2009b), with Kane (1979) being the first to identify the significance of fatty acids in this context. Interestingly, it appears that fatty acid oxidation in the oocyte and early embryo is closely intertwined with glucose metabolism, likely operating in an interdependent and compensatory manner (Sutton-McDowall et al., 2012; Paczkowski et al., 2014; Herrick et al., 2020). Amino acids also contribute to preimplantation embryo metabolism. The uptake and synthesis of amino acids have been observed in embryos across various species and have been linked to outcomes such as DNA damage, ploidy, embryo sex, and quality (Sturmey et al., 2009a; Picton et al., 2010). Supplementation with a combination of essential and non-essential amino acids in the absence of coculture was found to be advantageous for embryo development (Rosenkrans and First, 1994; Gardner et al., 1994; Steeves and Gardner, 1999), leading to an increase in the cell number of blastocysts cultured *in vitro*.

The effects of metabolic activity on the reduction-oxidation (REDOX) potential of the cell are now known to be as important, if not more so, than simple ATP production. Throughout the preimplantation phase, preserving a normal redox state is crucial for embryo development (Harvey et al., 2002). The cellular redox state is chiefly influenced by the ratios of key redox couples: NAD^+^:NADH (largely influenced by lactate dehydrogenase activity) and NADP^+^:NADPH (partially regulated by the pentose phosphate pathway (PPP)), along with the intracellular balance of reduced glutathione (GSH) to oxidized glutathione (GSSG). These nicotinamide molecules serve as vital cofactors for numerous metabolic reactions or act as their end-products. Thus, fostering optimal embryo metabolism in culture is imperative for preserving redox equilibrium and ensuring developmental competency.

## Oxygen

Although the culture medium plays a significant role in the quantity and quality of embryos produced, there are many other factors of the larger culture environment, such as air quality, plastics, oil, etc., that influence outcomes (Wale and Gardner, 2016). One of the most critical of these factors seems to be oxygen in the atmosphere within the incubator. In vivo, oxygen tension in the oviduct and uterus ranges from 1.5% to 8.7%, and is 5.3% at the time of blastocyst formation in hamsters and rabbits (Fischer and Bavister, 1993). Reduced concentrations of oxygen (<10%) relative to normal, atmospheric levels (20%) improve embryo development and viability in all species where it has been evaluated, including cattle (Thompson et al., 1990, 2000). As oxygen is necessary for mitochondrial ATP production, the benefits of reduced oxygen are associated with changes in metabolism, particularly an increase in glucose consumption and production of ATP via glycolysis (Thompson et al., 1996, 2000). However, oxygen can also influence the expression of genes not directly associated with metabolic activity, including anaphase promoting complex and myotrophin, suggesting beneficial effects of oxygen on other aspects of embryo physiology (Harvey et al., 2007).

## Growth factors

The fluids of the reproductive tract are known to contain a variety of growth factors, cytokines, and other cell signaling molecules, collectively known as embryokines, that influence embryo development and differentiation (Hansen and Tribulo, 2019). However, most culture media do not contain these embryokines. One reason for this discrepancy is the difficulty in studying the effects of growth factors. There are a large number of candidates whose effects can be inconsistent and dependent on the concentration used, the time of culture when they are included, and the composition of the medium (Herrick et al., 2018; Hansen and Tribulo, 2019; Amaral et al., 2022). In addition, the effects of these embryokines are often very subtle, difficult to interpret, and/or only apparent post-transfer (Hansen and Tribulo, 2019 ; Sang et al., 2020). For example, the inclusion of interleukin-8 (IL-8) in the culture medium increases the proportion of embryos that hatch but decreases the number of cells allocated to the inner cell mass (Sang et al., 2020). The effects of colony stimulating factor (CSF) 2 are dependent on the sex of the embryo, further complicating the formulation of media for typical (non-sorted sperm) IVF-produced embryos (Siqueira and Hansen, 2016). Although the addition of embryokines to culture media is not well understood, embryos do secrete them into the culture medium and these secreted embryokines are often credited with enhancing development of embryos cultured in groups versus individually.

## Fetal bovine serum

Fetal bovine serum remains a common supplement used to compensate for suboptimal embryo culture environments. Serum can buffer stressors and insults in the embryo culture system that may inhibit embryo development. Although the inclusion of serum in the culture medium can enhance development to the blastocyst stage, it may also diminish the ability of resulting embryos to be cryopreserved, and to establish and maintain pregnancy (Rizos et al., 2003; Amaral et al., 2022). Inclusion of serum in culture medium has also been implicated in large, or abnormal, offspring syndrome, although a direct causal link has not been confirmed (Lazzari et al., 2002; Hansen, 2020) nor is the timing of exposure or the threshold concentration of FBS leading to these effects understood. This congenital overgrowth syndrome is observed in ruminants born through assisted reproduction and characterized by significant dysregulation of the epigenome and transcriptome, excessive somatic growth, and various developmental anomalies such as enlarged tongues, umbilical hernias, muscle and skeletal deformities, abnormal organ growth, and aberrant placental development (Li et al., 2019, 2022; Rivera, 2019). The frequency of this syndrome may vary depending on the embryo culture system utilized. Although there are no good data on frequency of occurrence in the ET industry, rough estimates are in the 3-5% range. For producers using embryo transfer of *in vitro* produced bovine embryos on a large scale, this is a significant drawback and has been detrimental to the acceptance of this technology.

## Development of next generation embryo culture media

Investigations in our laboratory employed a gas chromatography-mass spectrometry platform to examine the nutrient composition of media following culture of individual embryos to better understand the metabolic profile of embryos *in vitro*. These metabolomic analyses suggested that embryos utilize only a fraction of the nutrients provided to them in the culture environment (Krisher et al., 2015; Herrick et al., 2016). The minimal amount of nutrients that are consumed compared to the abundance of nutrients available in the culture system prompted us to hypothesize that nutrient concentrations in the culture medium could be substantially decreased while still sustaining embryo development. In the mouse, nutrient concentrations (carbohydrates, amino acids, and vitamins) during the culture of murine embryos could be reduced by half with minimal impact on embryo development. However, decreasing nutrients, especially pyruvate and lactate, by more than 50% significantly impaired embryo development and viability (Ermisch et al., 2020). In the bovine embryo, development was largely unaffected when nutrient concentrations were reduced by as much as 75%, and some embryos were able to develop in medium containing only 6.25% of the original nutrient concentrations (Herrick et al., 2020). Other studies have replicated this work, demonstrating improved blastocyst development and quality when nutrients were reduced by half (Santos et al., 2021). The exceptional resilience of the bovine embryo to significant reductions in nutrient availability is linked to its capacity to utilize endogenous lipids. To further refine our reduced nutrient concentration media for bovine embryos, we supplemented this media with exogenous lipids and L-carnitine to promote lipid metabolism. Under these conditions, blastocyst development was significantly improved, and the expression of embryo quality related genes was increased, although blastocyst cell number was lower (Pasquariello et al., 2023).

Transcriptomic analysis of *in vivo* produced embryos compared to *in vitro* embryos cultured in either standard or reduced nutrient conditions demonstrated that *in vitro* embryos produced in standard conditions were more active metabolically compared to *in vivo* produced embryos, while metabolic processes were in fact downregulated in embryos developed under reduced nutrient conditions (Ming et al., 2023). Embryos developed under reduced nutrient conditions upregulated genes associated with protein hydrolysis and cell survival, a strategy to maintain cellular homeostasis that is reminiscent of the high protein turnover that limits oxidative damage and extends lifespan in caloric restriction. Embryos developed in reduced nutrient conditions also had increased transmembrane transport, likely necessary for nutrient uptake in a restricted environment, again similar to caloric restriction response. Overall, the developmental potential of embryos cultured in reduced nutrient conditions was closer to *in vivo* embryos than that of embryos cultured *in vitro* under standard conditions (Ming et al., 2023). These studies open a new frontier in bovine embryo culture media development and may result in a more developmentally competent *in vitro* embryo. However, additional studies including post-transfer embryo viability and calf health are needed to fully appreciate the capability of this reduced nutrient culture system.

## What does the future hold for bovine embryos?

We might take a cue from the world of human IVF. Embryo diagnostics, such as embryo sex, genotype, identification of chromosomal abnormalities, presence of desirable production traits, and or prediction of viability would add significant value to a bovine embryo (Figure 1). Today, we can successfully genotype and sex embryos by taking a biopsy of the trophectoderm at the blastocyst stage without significantly compromising subsequent development (Fujii et al., 2019; Oliveira et al., 2023). In fact, biopsy is a recognized emerging technology as the number of embryos being sexed or genotyped is now being tracked by the IETS Data Retrieval Committee and was greater than 23,000 embryos in 2022. Bovine embryos can also be screened for chromosomal abnormalities via trophectoderm biopsy (Turner et al., 2019; Silvestri et al., 2021). Biopsy procedures necessitate advanced technical expertise and expensive equipment, potentially influencing both the precision of genetic testing and implantation potential. Reliable genotype information can be obtained from embryo biopsies, but only a limited number of laboratories are currently using this technology due to these limitations. Prediction of pregnancy success in human embryos using artificial intelligence and machine learning with either photos or time lapse video is in use today (Vermilyea et al., 2020; Enatsu et al., 2022; Diakiw et al., 2022; Salih et al., 2023). These algorithms haven't yet been developed specifically for cattle embryos, partly because of the high cost of time lapse incubators. However, affordable models targeted at the veterinary sector are emerging, suggesting this could soon become a possibility for bovine IVF, and preliminary research supports this assumption (Sugimura et al., 2017). Alternatively, information could be gained about embryo genetics and viability non-invasively from cell free DNA or extracellular vesicles in the culture medium after blastocyst development, or from blastocoel fluid. Noninvasive preimplantation genetic testing (niPGT) of human embryos demonstrates that cell free DNA suitable for genetic analysis can routinely be obtained from these samples, offering an alternative to embryo biopsy that requires less skill, poses less risk to the embryo, and is less expensive (Huang et al., 2019; Rubio et al., 2019; Leaver and Wells, 2020; Rubio et al., 2020; del Collado et al., 2023). However, reported concordance rates between cell free DNA and biopsy results are variable between studies, and the diagnostic value of noninvasive preimplantation genetic testing remains controversial in human IVF (Huang et al., 2023; Lledo et al., 2023).

Bovine embryos can also be a source of information to discover regulatory pathways important for development. Extracellular vesicles and their microRNA and protein cargo have emerged as pivotal bi-directional messengers between the embryo and its environment at various stages of pre- and post-implantation development (Lange-Consiglio et al., 2020; Salilew-Wondim et al., 2020; Tesfaye et al., 2020; Guzewska et al., 2023). Extracellular vesicles can influence blastocyst development and modulate embryo stress in vitro, potentially by modulating embryo gene expression (Alminana et al., 2017; Lopera-Vasquez et al., 2017; Menjivar et al., 2023). In addition, an embryos’ extracellular vesicles and microRNAs may provide clues about the quality and viability of that embryo, possibly leading to non-invasive diagnostic assays (Marin and Scott, 2018; Cimadomo et al., 2019; Hawke et al., 2021). Finally, extended embryo culture (Shahbazi et al., 2016; Isaac et al., 2024) and synthetic embryo models (Kagawa et al., 2022; Kim et al., 2023; Pinzon-Arteaga et al., 2023; Yu et al., 2023) enable us to discover physiological processes occurring in the embryo after the blastocyst stage, a period difficult to study in vivo. In the long run, delivering embryos at a reduced cost (closer to that of a straw of sexed semen, perhaps) while maintaining high value and enhancing outcomes will necessitate concurrent advancements in multiple areas. These include the development of improved culture media supporting normal embryo physiology, enhancements in the culture environment, and the integration of various diagnostic technologies. The forthcoming decade promises to be an exciting era of exploration in bovine *in vitro* embryo production and diagnostics. As these breakthroughs are applied to the commercial bovine embryo transfer industry, they will further bolster the utilization of *in vitro* produced embryos and propel the industry towards a more sustainable approach to feeding the world. However, it's imperative that these advancements are accessible at a price point that ensures a reasonable return on investment for producers, for this technology to be truly transformative.

## Conclusion

Rapid adoption of *in vitro* embryo production in the bovine industry highlights the importance of embryo technology for genetic improvement. A growing human population, food insecurity, and climate change put enormous pressure on producers to make protein production more efficient and sustainable. However, large inefficiencies in the system significantly increase cost and may limit full realization of the potential of the technology. The low conversion percentage of oocytes to blastocysts, and the quality of those blastocysts produced, significantly contribute to these inefficiencies. Improvements in embryo culture media resulting in a more viable, more freezable embryo capable of a high level of pregnancy establishment and maintenance, and normal healthy calf production, is essential.
